# When do bystanders get help from teachers or friends? Age and group membership matter when indirectly challenging social exclusion

**DOI:** 10.3389/fpsyg.2022.833589

**Published:** 2022-08-30

**Authors:** Ayşe Şule Yüksel, Sally B. Palmer, Eirini Ketzitzidou Argyri, Adam Rutland

**Affiliations:** ^1^Department of Psychology, University of Exeter, Exeter, United Kingdom; ^2^Graduate School of Education, University of Exeter, Exeter, United Kingdom

**Keywords:** indirect bystander reactions, social and moral reasoning, children, adolescents, group membership

## Abstract

We examined developmental changes in British children’s (8- to 10-year-olds) and adolescents’ (13- to 15-year-olds, *N* = 340; Female *N* = 171, 50.3%) indirect bystander reactions (i.e., judgments about whether to get help and from whom when witnessing social exclusion) and their social-moral reasoning regarding their reactions to social exclusion. We also explored, for the first time, how the group membership of the excluder and victim affect participants’ reactions. Participants read a hypothetical scenario in which they witnessed a peer being excluded from a school club by another peer. We manipulated the group membership of the victim (either British or an immigrant) and the group membership of the excluder (either British or an immigrant). Participants’ likelihood of indirect bystander reactions decreased from childhood into adolescence. Children were more likely to get help from a teacher or an adult than getting help from a friend, whereas adolescents were more likely to get help from a friend than getting help from a teacher or an adult. For both indirect bystander reactions, children justified their likelihood of responding by referring to their trust in their teachers and friends. Adolescents were more likely to refer to group loyalty and dynamics, and psychological reasons. The findings support and extend the Social Reasoning Developmental (SRD) approach by showing the importance of group processes with age in shaping children’s judgments about how to respond indirectly by asking for help from others, when they are bystanders in a situation that involves exclusion. The findings have practical implications for combating social exclusion and promoting prosocial bystander behavior in schools.

## Introduction

Social exclusion involves being left out of a group or an activity and has many long-term detrimental psychological and academic effects on children (Buhs et al., 2006; Gazelle and Druhen, 2009; Lansu et al., 2017). When peers intervene to challenge social exclusion as bystanders (i.e., witnesses), their reactions can help to reduce exclusion (Polanin et al., 2012; Evans et al., 2014; Palmer and Abbott, 2018). However, bystander reactions can be either *direct* (i.e., intervening to stop the incident by confronting the perpetrator) or *indirect* (i.e., getting help from a teacher or friend; Pronk et al., 2013; Lambe and Craig, 2020). Unlike direct forms, indirect bystander reactions to challenge bullying arguably require less resources (i.e., cognitive empathy, self-efficacy) and involve less risks (i.e., potential retaliation by the bully, perceived costs within the peer group; Levy and Gumpel, 2018; Lambe et al., 2019). Therefore, when bystanders witness social exclusion, indirect challenging may be more likely than direct challenging. Indirect bystander reactions in a school context can involve getting help from either a teacher/other adult or a friend within the peer group, yet we know little about developmental and contextual effects on indirect bystander reactions. This study examines age differences in terms of how children and adolescents indirectly challenge exclusion as bystanders, and whether such indirect challenging is dependent on the immigrant status of the excluder and the victim.

The present study examined age differences in British children’s and adolescents’ indirect prosocial bystander reactions to social exclusion using hypothetical scenarios. We manipulated both the group membership of the excluder and the group membership of the victim. Participants read a scenario in which either a British or an immigrant peer was excluded from a school club by either a British or an immigrant peer, and answered questions measuring their likelihood of indirect bystander reactions (i.e., getting help from a teacher or an adult and getting help from a friend). This study explored the immigrant context as it is becoming more relevant in today’s global world where immigrant children and adolescents experience pervasive social exclusion and discrimination in school settings (Stevens et al., 2020; Xu et al., 2020). This bias-based form of exclusion stems from prejudice and discrimination and can have more negative health and academic consequences than interpersonal forms of exclusion (Oxman-Martinez et al., 2012; Killen et al., 2013; Brown and Lee, 2015). A better understanding of developmental and contextual effects on indirect bystander challenging can inform anti-bullying programs designed to improve prosocial bystander behavior among students and can have a crucial role in combating the social exclusion of immigrants in schools (Polanin et al., 2012; Gönültaş and Mulvey, 2019).

### Social reasoning developmental perspective on social exclusion

Our research was guided by the Social Reasoning Developmental approach (SRD, Rutland et al., 2010; Killen and Rutland, 2011; Rutland and Killen, 2015), which provides a developmental intergroup framework to examine social exclusion in childhood by drawing upon different theories and research (i.e., social identity theory and social domain theory; Turiel, 1983, 2008; Tajfel and Turner, 1986; Nesdale, 2004). The Social Reasoning Developmental approach highlights the interplay between moral decision-making and intergroup factors such as group membership and group dynamics in understanding children’s and adolescents’ bystander reactions to social exclusion (Palmer and Abbott, 2018; Palmer et al., 2021).

Only a few studies conducted in North America drawing from the Social Reasoning Developmental approach have explored indirect bystander reactions using hypothetical scenarios (Mulvey et al., 2019, 2020a; Gönültaş and Mulvey, 2020; Knox et al., 2021). Most of these studies measured indirect bystander reactions but generated composite measures of general bystander reactions including direct and indirect measures together (Mulvey et al., 2019, 2020a; Knox et al., 2021). Only one study used a separate measure of indirect bystander reactions and found that younger European American adolescents (mean age 12 years) were more likely to report that they would get help from others (i.e., a composite variable of getting help from teachers and adults and getting help from peers) compared to older adolescents (mean age 15 years) when they witnessed peer aggression (Gönültaş and Mulvey, 2020). What is not known, however, is whether there are any developmental trends in indirect bystander reactions from childhood into adolescence, especially in the context of social exclusion. Getting help as a bystander is a very important way of addressing biased-based social exclusion because it identifies the behavior, often publicly, in a way that can change group norms, and potentially provide a path to less such exclusion in the future. This is especially so for children, who may have less power than adults, so getting others involved may be necessary to change these types of social exclusion. Social exclusion is conceptually different from other forms of bullying, such as aggression which is perceived as a moral transgression (i.e., harmful to the welfare of the victim). Social exclusion is not always considered immoral and is often legitimized in order to maintain group identity, group norms or group functioning (Killen and Rutland, 2011).

A decline in indirect challenging of social exclusion in a peer group context would be expected according to the Social Reasoning Developmental approach since it emphasizes how group context and dynamics play an increasing role in the shift from childhood to adolescence, affecting potential bystander reactions to social exclusion (i.e., “how would the group react to me telling a teacher” or “instead should I tell a friend”?; Killen and Rutland, 2011; Palmer et al., 2021). Studies have shown that, from an early age, children start to understand social mechanisms and become aware of group life (Smetana, 2006). They start to affiliate with groups, develop group identities, and show ingroup bias and loyalty toward ingroups (Nesdale, 2004; Dunham et al., 2011; Misch et al., 2016). With age, and into later childhood and adolescence, an advanced understanding of group identity and group loyalty emerges (Horn, 2003; Abrams and Rutland, 2008), with a better understanding that being seen as disloyal to the group can have consequences and can lead to the disloyal member being excluded from the peer group (Mulvey and Killen, 2015; Mulvey et al., 2016). Thus they become more likely to show group loyalty and ingroup bias when evaluating their peers and determining their bystander reactions to exclusion. Research shows that with age, children can support negative acts when they think that their peer group is okay with that act (Nipedal et al., 2010; Mulvey et al., 2016). In the current study, therefore, we expected that adolescents would be less likely to report indirect bystander reactions (i.e., getting help from a teacher or an adult and getting help from a friend).

### Different forms of indirect bystander reactions

Studies using the Social Reasoning Developmental approach to examine bystander reactions, to date, have not typically explored separately the bystander reactions of getting help from a teacher and getting help from a friend. They have usually combined various bystander reaction items to create composite variables, including the reactions of getting help from a teacher and a friend in different categories such as inactive bystander responses (e.g., Mulvey et al., 2019, 2020a; Gönültaş et al., 2020) or seeking help responses (e.g., Gönültaş and Mulvey, 2020) or bystander intention/intervention (e.g., Palmer et al., 2015, 2022; Knox et al., 2021). Examining the indirect reactions of getting help from a teacher and getting help from a friend separately is crucial. From late childhood into adolescence there is increasing focus on group identity and loyalty within the peer group (Mulvey and Killen, 2015; Mulvey et al., 2016). Because of this, children and adolescents may reason differently about who they would get help from (i.e., teachers or friends within a peer group). Engaging in these two indirect forms of prosocial bystander reactions may have potentially different perceived group consequences for children and adolescents (i.e., how they think they may be perceived within their peer group). This could make adolescents, relative to those in late childhood, more likely to engage in indirect bystander reactions involving peers rather than teachers. In the current study, for the first time, we focused on these two types of indirect bystander reactions to social exclusion: getting help from a teacher or an adult (1) and getting help from a friend (2).

### Getting help from a teacher

Teachers have a critically important role in combating bullying, including social exclusion (Brendgen and Troop-Gordon, 2015) and they are usually the first adults to respond to conflicts among peers. However, to respond to bullying incidents, teachers first need to know about bullying incidents. Research shows that teachers are not present at most bullying incidents (Ozada Nazim and Duyan, 2021). When they are present, they take action in only 4% of bullying episodes in the playground (Craig and Pepler, 1997) and 18% when bullying incidents happen in the classroom (Atlas and Pepler, 1998). Their lack of action can be related to them not being aware of bullying or not observing the bullying incidents in person (Craig et al., 2000). Research also shows that teachers do not perceive themselves as prepared to identify bullying because of a lack of awareness and training (Bauman and Hurley, 2005; Beran, 2005; Novick and Isaacs, 2010). Their likelihood of reacting can also be impacted by the type of bullying. While teachers easily identify physical forms as bullying, they can think nonphysical forms of bullying (e.g., social exclusion) are less harmful and less serious than physical and verbal forms (Yoon and Kerber, 2003; Bauman and Del Rio, 2006) and some do not consider them as bullying at all (Boulton, 1997; Craig et al., 2000). Moreover, one piece of research showed that even when teachers were aware of bullying, they preferred not to intervene in 25% of bullying incidents (Atlas and Pepler, 1998). Other research showed that teachers were less likely to identify bullying among secondary school adolescents than among elementary school children (Leff et al., 1999).

One way to make teachers take action is students who are often bystanders to bullying incidents (e.g., social exclusion) telling them about bullying. Research found that the strongest predictor of teacher intervention was students telling them about bullying incidents compared to the other forms (i.e., observing bullying with their own eyes; Novick and Isaacs, 2010). Another study showed the more children reported bullying to their teachers, the lower the levels of victimization were observed (Cortes and Kochenderfer-Ladd, 2014). However, children do not often tell their teachers about bullying incidents and they become less likely to inform a teacher as they become adolescents (Smith and Shu, 2000).

### Getting help from a friend

Another form of indirect bystander reaction is getting help from a friend. This is an important response because it increases the likelihood of further bystander intervention by another peer. Indeed, research shows that being asked by a victim to help a victim makes that individual more likely to intervene themselves (Machackova et al., 2018). Bullying research, however, mainly focuses on victims getting help from a friend, but not on bystanders getting help from a friend. Research also shows that victims of bullying are more likely to tell a friend than to tell a teacher (Smith and Shu, 2000; Blomqvist et al., 2020) and although their likelihood of telling a teacher decreases with age, the likelihood of telling a friend remains high as it is perceived to be less risky (Oliver and Candappa, 2007). This is in line with the Social Reasoning Developmental approach, as with an increasing understanding of group dynamics (i.e., group repercussions), adolescents develop the ability to evaluate the consequences of challenging groups (Mulvey and Killen, 2016, 2017). Although victims’ perspectives can give an insight into how they perceive getting help from a friend, examining bystanders’ perspectives is also important since if the bystander asks a friend for help when they witness exclusion this can increase the likelihood of victims getting help. However, no studies have yet explored the “getting help from a friend” bystander reaction specifically. In the current study, we expected that children would be more likely to get help from a teacher or an adult than from a friend when they witnessed social exclusion. With increasing recognition of the social consequences and risks (Oliver and Candappa, 2007; Mulvey et al., 2016), adolescents would be more likely to get help from a friend than to get help from a teacher or an adult.

### Group membership of excluder and victim

The social reasoning developmental model of social exclusion would also anticipate that the group membership of the excluder and victim is related to whether children and adolescents as bystanders get help from either a teacher/adult or a friend. Previous developmental research has examined children’s evaluations of aggressors who either shared or did not share group membership with the children (Nesdale et al., 2013) and found that children were more positive toward aggressors who belonged to the same group as them. This suggests that when the excluder is an ingroup compared to an outgroup peer, youth should be especially concerned about the consequences of telling a teacher. This is because it may affect their position in the group, since the act of telling a teacher may be seen as disloyal. This could consequently lead to them being excluded from their peer group or at least fearing this outcome (Mulvey and Killen, 2015; Mulvey et al., 2016).

Developmental research also suggests that the group membership of the victim relates to whether youth indirectly challenge social exclusion. For example, Gönültaş and Mulvey (2020) found that adolescents were more likely to get help from a teacher or friend when the victim was an ingroup peer compared to an outgroup peer. In the current study, for the first time, the group membership of the victim (either British or an immigrant peer) and the group membership of the excluder (either British or an immigrant peer) were manipulated in a fully crossed design (i.e., a British peer excluding an immigrant victim, an immigrant peer excluding an immigrant victim, a British peer excluding a British victim, or an immigrant peer excluding an immigrant peer). We expected that when the excluder was an ingroup compared to an outgroup peer, participants would be less likely to report indirect prosocial bystander reactions. Additionally, when the victim was an ingroup compared to an outgroup peer, participants should be more likely to report indirect prosocial bystander reactions.

### Social and moral reasoning

In addition to examining the developmental and contextual differences in indirect prosocial bystander reactions, the current study examined how children and adolescents justified their likelihood of getting help from a teacher and getting help from a friend to provide more insight into developmental differences. Participants’ reasoning was coded using categories from Social Domain Theory (Turiel, 2008; Smetana, 2013) and previous research that draws from the Social Reasoning Developmental approach to social exclusion and bystander responses (e.g., Killen et al., 2013; Palmer et al., 2015; Rutland et al., 2015; Mulvey et al., 2016). The Social Reasoning Developmental approach indicates that children and adolescents attempt to balance different concerns in different domains of knowledge when making decisions about bystander responses (Killen and Rutland, 2011; Palmer et al., 2015; Mulvey et al., 2016). In line with the Social Domain Theory, the Social Reasoning Developmental approach contends that children draw on three domains of knowledge—moral concerns (fair and equal treatment of others), social-conventional or social group concerns (traditional beliefs, group identity and group functioning) and psychological concerns (autonomy and personal preferences)—when evaluating social exclusion and bystander reactions (Killen et al., 2013; Palmer et al., 2015). It is worth noting that other theoretical approaches, for example, Moral Foundation Theory (Haidt and Graham, 2007), contends affinity to one’s social group is a moral concern, and this issue is a topic of debate (see Haste, 2013; Harper and Rhodes, 2021).

Which domains are prioritized alternates as children’s comprehension of intergroup relations and group dynamics increases with age. At an early age, children often regard exclusion as wrong and reject it due to moral concerns about fairness, equal treatment, and psychological harm, thereby applying basic moral principles to situations (Killen et al., 2001; Rutland and Killen, 2015). With age, however, they often find exclusion relatively acceptable due to having socio-conventional concerns (i.e., group membership, group dynamics, group functioning, and group loyalty) and psychological concerns (i.e., autonomy, and personal choice, Horn, 2008; Killen et al., 2013; Rutland and Killen, 2015). For example, previous research showed that 10th grade participants were more likely to refer to group loyalty to justify their decision about peer group dynamics compared to 8th graders (Rutland et al., 2015). A similar pattern has been observed in the context of bystander reactions. Research has shown that children tend to use more social-conventional and psychological reasons while justifying their likelihood of bystander challenging with age (Palmer et al., 2015; Mulvey et al., 2016). For example, one piece of research showed that children used moral reasoning more than adolescents did, whereas adolescents used psychological reasoning more than children did while justifying their prosocial bystander intentions (Palmer et al., 2015). Given these findings, it was expected that children would use moral reasoning more when justifying their likelihood of indirect bystander reactions to social exclusion whereas adolescents would use social-conventional and personal reasoning more.

### The present study

The main aim of this study was to explore developmental differences in children’s and adolescents’ indirect bystander reactions and how they reasoned about them. We focused on two forms of indirect bystander reactions—(1) getting help from a teacher and (2) getting help from a friend. We also explored contextual effects, by examining whether the group membership of the excluder and the group membership of the victim had an influence on their indirect bystander reactions by manipulating the excluder’s membership (i.e., British or an immigrant peer) and the victim’s membership (i.e., British or an immigrant peer). We focused on two age groups and compared children’s and adolescents’ indirect bystander reactions as previous research has shown a developmental shift from childhood into adolescence whereby, compared to children, adolescents are more likely to evaluate social exclusion focusing more on group-related concerns (Killen et al., 2013; Mulvey et al., 2014). Furthermore, previous research has shown a developmental shift between these two age groups with adolescents’ greater understanding of group dynamics and intergroup factors suggesting that they are less likely to show bystander intervention in peer group contexts (Palmer et al., 2015; Mulvey et al., 2016).

Research has also shown that adolescents’ bystander challenging toward outgroup members can increase when they have high levels of intergroup contact (Abbott and Cameron, 2014). When children have higher levels of intergroup contact, they can be less likely to be prejudiced against those groups, i.e., immigrants (Titzmann et al., 2015) and their evaluations regarding exclusion can become more positive (Crystal et al., 2008; Park et al., 2019). In the current study, therefore, we measured participants’ intergroup contact with immigrants in order to use this as a covariate.

### Hypotheses

Based on the theoretical framework, i.e., the Social Reasoning Developmental model, and developmental research, we tested four hypotheses in this study.

*Hypothesis 1*: Adolescents would be less likely to report indirect bystander reactions to social exclusion as bystanders compared to children.

*Hypothesis 2*: Children would be more likely to get help from a teacher or an adult than from a friend when they witnessed social exclusion as bystanders. Meanwhile, adolescents would be more likely to get help from a friend than getting help from a teacher or an adult as bystanders.

*Hypothesis 3*: When the excluder was an ingroup compared to an outgroup peer, youth would be less likely to report indirect bystander reactions to social exclusion. When the victim was an ingroup compared to an outgroup peer, youth would be more likely to report indirect bystander reactions to social exclusion.

*Hypothesis 4*: Children would use moral reasoning more when justifying their likelihood of indirect bystander reaction to challenge exclusion when witnessing social exclusion whereas adolescents would use social-conventional and personal reasoning more. It was an open question as to whether social and moral reasoning would vary depending on the group membership of the victim or the excluder.

## Materials and methods

### Design

The present study adopted a 2 (Age Group: children, adolescents) × 2 (Excluder Membership: British, immigrant) × 2 (Victim Membership: British, immigrant) × 2 (Indirect Bystander Reactions: getting help from a teacher or an adult and getting help from a friend) mixed experimental design (see Table 1). Participants were randomly presented with a scenario in which either a British or an immigrant peer excluded either a British or an immigrant victim from a school club. The dependent variables were participants’ likelihood of engaging in two indirect forms of bystander reactions: (1) getting help from a teacher or an adult and (2) getting help from a friend, and (3) participants’ social and moral reasoning for these two bystander reactions.

**Table 1:** The study design.

Condition	Excluder membership	Victim membership
1	British	British
2	British	Immigrant
3	Immigrant	British
4	Immigrant	Immigrant

### Participants

The participants were 424 British children and adolescents from two age groups: children (*N* = 205, 48.3%, range = 8–10 years, *M*_age_ = 9.03, SD = 0.74) and adolescents (*N* = 219, 51.7%, range = 13–15 years, *M*_age_ = 13.44, SD = 0.63), evenly distributed across gender groups (Female *N* = 209, 49.3%). Participants were asked if they were British or immigrants. Participants who identified themselves as immigrants (*N* = 84) were excluded from the final analyses. A final sample of 340 participants (children, *N* = 155, *M*_age_ = 9.05, SD = 0.74; adolescents, *N* = 185, *M*_age_ = 13.49, SD = 0.65; Female, *N* = 171, 50.3%) was analyzed.

The present study was carried out in diverse areas of a large city in south-eastern England where participants were from lower to middle-class socioeconomic status groups. The final sample included 24.7% South Asian British, 17.6% White British, 17.1% Black British, 12.1% Dual-Heritage, 9.7% European British and 6.5% other (including Arab, Japanese British), with 12.4% of the sample withholding their ethnic identity information. Power analysis for an analysis of variance with three factors and eight groups was conducted in G*Power to determine a sufficient sample size using an Alpha level of 0.05, power of 0.95, and a small to medium effect size of 0.25 (Faul et al., 2007). The required sample size for this study was 279.

### Procedure

All participants received parental consent and gave assent. They completed the assessment on individual computers using the experimental software Qualtrics, in their school under the guidance of the researcher and were debriefed at the end. Participants were asked to imagine that they were part of a gender-matched group; the “British group of friends” (e.g., Killen et al., 2013; Mulvey et al., 2016; Mulvey and Killen, 2016). Following the conventions of the minimal group paradigm (Nesdale, 2008), in order to enhance identification with the group, participants were asked to select a name and a symbol for their group. Next, participants were asked to imagine another group of friends, i.e., the “immigrant group of friends.” In line with previous studies involving children (Cameron et al., 2006; Abbott and Cameron, 2014), participants were presented with the following definition of immigrants: “*immigrants are individuals who live in Britain but are not British since they were born in and came from other countries.*”

#### Social exclusion scenario

Next, participants read a hypothetical scenario in which either a British or an immigrant peer was excluded from a cooking club by either a British or an immigrant peer. The reason for the exclusion was ambiguous as in real-life situations, excluders do not always express the reason behind excluding their victims explicitly. It is not always clear that exclusion is biased-based bullying, and it is a developmental challenge for children to determine whether intergroup exclusion is based on prejudice and discrimination (Killen and Rutland, 2011).

An example scenario of when the group membership of the excluder was British and the group membership of the victim was immigrant is as follows: “*Imagine that your group, the British group of friends, decide to form a cooking club for students who like cooking British food in your school. [Victim] from the immigrant group of friends likes cooking British food and wants to join the cooking club. [Excluder], from your group, does not want him/her to join the cooking club. [Excluder] shares his/her opinion with the others in the club and they agree to leave [victim] out.”*

### Indirect bystander reaction measures

#### Getting help from a teacher or an adult

To measure participants’ likelihood of getting help from a teacher or an adult as a bystander, participants were asked: “How likely or not likely is it that you would get help from a teacher or an adult?” and responded on a 1 (really not likely) to 6 (really likely) scale (adapted from Mulvey et al., 2016; Gönültaş and Mulvey, 2020).

#### Getting help from a friend

To measure participants’ likelihood of getting help from a friend as a bystander, participants were asked: “How likely or not likely is it that you would get help from a friend?” and responded on a 1 (really not likely) to 6 (really likely) scale (adapted from Mulvey et al., 2016; Gönültaş and Mulvey, 2020).

#### Reasoning justifications

Participants also justified their indirect bystander reactions in open-ended “why?” questions following the likelihood measures. The responses to the reasoning questions were analyzed using a coding system drawing from Social Domain Theory (Turiel, 1983; Smetana, 2006; Smetana et al., 2014), and prior research on social exclusion and bystander responses (Killen and Stangor, 2001; Killen et al., 2002, 2013; Palmer et al., 2015). The responses were coded under three general domains: moral, social-conventional or group and psychological concerns. The moral concerns included references to fairness, individual rights and welfare; the social conventional or group concerns included references to trust in teachers and friends, mistrust in teachers and friends, group dynamics and loyalty. The psychological domain included references to autonomy, personal preferences and personal characteristics. Consequently, five categories that fell under three general domains were created: one moral category, three social-conventional categories and one psychological category (see Table 2).

**Table 2:** Coding domains, categories, content, and example items.

Domain	Categories	Content	Example items
**Moral**		Fairness and individual rights	“That not fair” “He does not deserve to be out” “Because it is not right to leave a child out”
		Welfare	“I do not want him to be alone” “So she feels included”
**Social-conventional**	Trust in teachers/friends	Trust in teachers/adults	“Because teachers help you and if somebody is left out you can tell them and they fix it” “Teachers are trust-able”
		Trust in friends	“A friend will sort the problem out” “Friends are reliable”
	Mistrust in teachers/friends	Mistrust in teachers/adults	“Teachers do not care most of the time” “They would not understand and might take it the wrong way”
		Mistrust in friends	“They cannot help this situation” “They will not care”
	Group Dynamics/ Loyalty	Understanding of group dynamics	“Because we all voted that we should kick him out” “It’s the friend groups problem and it is not a big of a deal so they should sort it out themselves” “Because I’d think that we can work it out ourselves”
		Group loyalty and repercussions	“I would not snitch” “As I would not want my friends getting in trouble, I ain’t a snake”
**Psychological**		Autonomy	“I am capable of doing it myself” “Because if I was in that situation I would not want anyone else involved”
		Personal preferences/characteristics	“There is no point” “It is not big of a deal” “I am not very confident”
**Undifferentiated**			“I do not know” “Not sure”

The moral domain categories and one of the social-conventional categories (mistrust in teachers/friends) were removed from the reasoning analyses as they were used less than 10% for both getting help from a teacher item (moral, 7.9%; trust in teachers, 21.2%; mistrust in teachers, 8.5%; group loyalty and dynamics, 11.5%, psychological, 15%; undifferentiated, 10.3%; missing, 25.6%) and getting help from a friend item (moral, 2.9%; trust in friends, 22.9%; mistrust in friends, 5.3%; group loyalty and dynamics, 12.4%, psychological, 16.5%, undifferentiated, 11.5%; missing, 28.5, see Table 3). Undifferentiated responses (i.e., uncodable statements) were omitted from the central analyses along with missing responses. Interrater reliability was conducted on 25% of each reasoning question by two coders one of whom was blind to the hypotheses of study and analyses of agreement revealed strong inter-rater reliability for both questions (getting help from a teacher or an adult, getting help from a friend, Cohen’s kappa = 0.86, 0.89, respectively).

**Table 3:** Categories used in reasoning analyses.

Measures	Moral domain	Social-conventional domain			Psychological domain
		Trust in teachers/friends	Mistrust in teachers/friends	Group dynamics and loyalty	
Getting help from a teacher or an adult	<10%	(1) Trust in teachers	<10%	(2) Group dynamics/loyalty	(3) Psychological
Getting help from a friend	<10%	(1) Trust in friends	<10%	(2) Group dynamics/loyalty	(3) Psychological

#### Intergroup contact

An adapted version of the intergroup contact measure developed by Crystal et al. (2008) was used to measure the level of intergroup contact with immigrants. The scale contained six items (e.g., how many students in your school are immigrants?). The responses to these items range from 1 (“none”) to 4 (“most”), *α* = 0.84.

### Plan of analyses

The analyses were conducted using SPSS 28. Initially, we conducted two separate linear regression analyses with two indirect bystander reactions as the dependent variables and age group, excluder membership, victim membership, gender and intergroup contact, as predictors. Intergroup contact and gender were not significant predictors, so they were dropped from subsequent analyses (see Supplementary materials).

The data was analyzed using a 2 (Age Group: children, adolescents) × 2 (Excluder membership: British, immigrant) × 2 (victim membership: British, immigrant) × 2 (Indirect Bystander Reaction: getting help from a teacher or an adult, getting help from a friend) ANOVA with repeated measures on the last factor. Follow up tests were performed using the Bonferroni correction to control for Type I errors. In line with the reasoning literature (e.g., McGuire et al., 2017), the reasoning responses were analyzed using multinomial logistic regression models. We modeled the effects of age group (children, adolescents), and excluder membership (British, immigrant) and victim membership (British, immigrant), across reasoning categories for each item.

## Results

### Indirect bystander reactions

**Figure 1:**
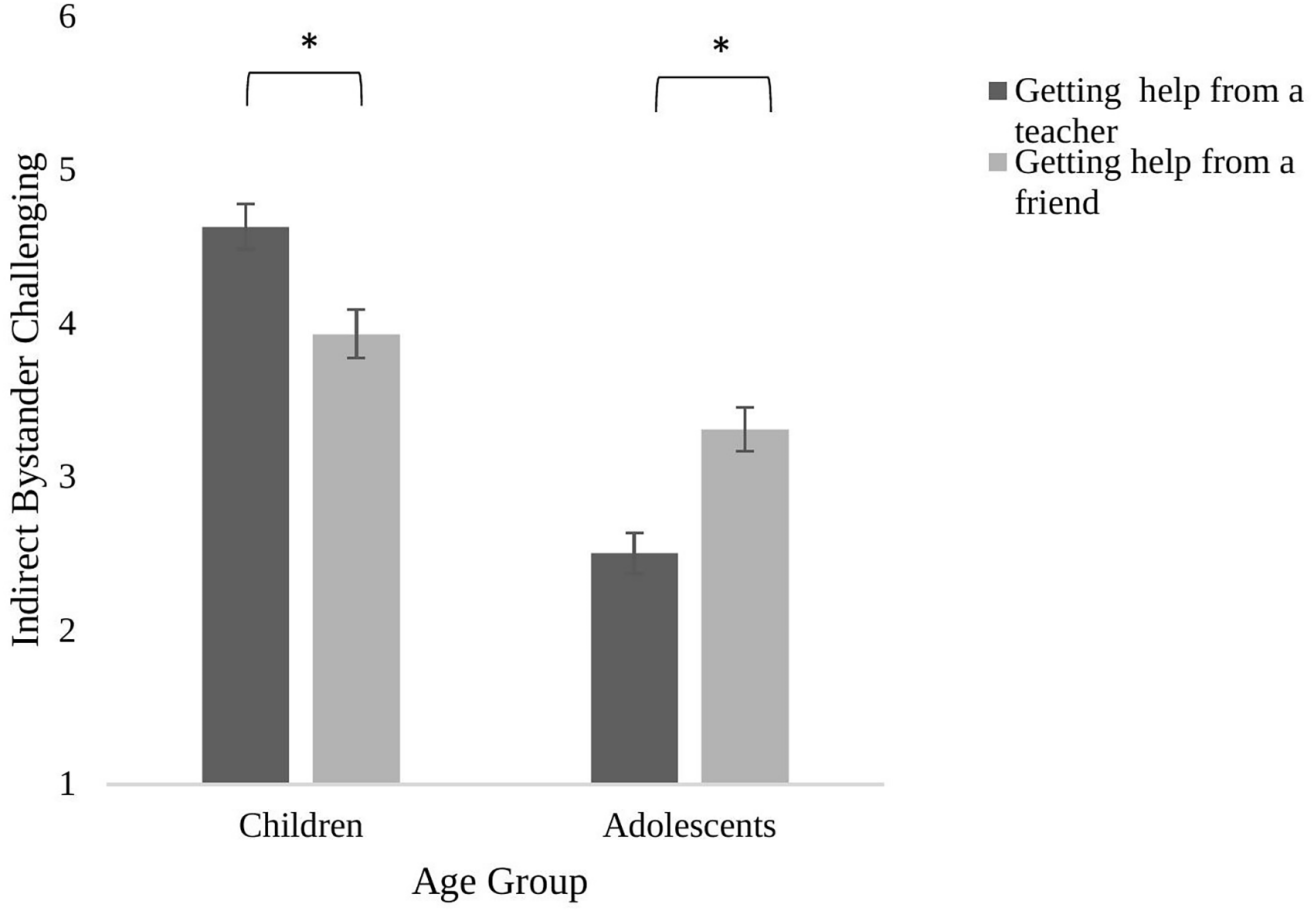
Participants’ indirect bystander challenging as a function of age group. Error bars show standard error. *p < 0.001.

Test of between participant factors revealed a significant main effect of age group on indirect bystander reactions, *F* (1, 285) = 68.44, *p* < 0.001, partial *η*^2^ = 0.194. As expected, in line with Hypothesis 1, children were more likely to report indirect bystander reactions (*M* = 4.28, *SD* = 1.90) compared to adolescents (*M* = 2.91, *SD* = 1.60). Test of within participants factors revealed a significant interaction between indirect bystander reactions and age group, *F* (1, 285) = 39.10, *p* < 0.001, partial *η*^2^ = 0.121. As anticipated, in line with hypothesis 2, pairwise comparisons showed that adolescents were less likely to get help from a teacher or an adult (*M* = 2.51, *SD* = 1.58) than getting help from a friend (*M* = 3.31, *SD* = 1.63, *p* < 0.001, partial *η*^2^ = 0.080). In contrast, children were more likely to get help from a teacher or an adult (*M* = 4.63, *SD* = 1.78) than getting help from a friend (*M* = 3.93, *SD* = 2.03, *p* < 0.001, partial *η*^2^ = 0.051, see Figure 1).

**Figure 2:**
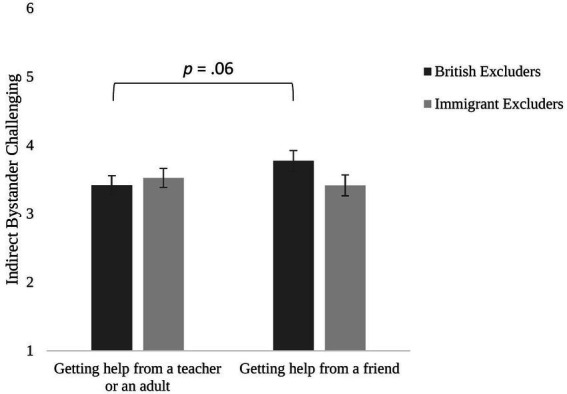
Participants’ indirect bystander challenging as a function of age group membership of the excluder. Error bars show standard error.

Hypothesis 3 was not fully supported since the test of between-participant factors did not show any main effect of the group membership of the excluder or the group membership of the victim, both *p*s > 0.05. However, our exploratory findings indicated an interaction between indirect bystander reactions and excluder membership, *F* (1, 285) = 4.70, *p* = 0.031, partial *η*^2^ = 0.016. Pairwise comparisons showed that when the excluder was British, participants were marginally less likely to get help from a teacher or an adult (*M* = 3.42, *SD* = 1.40) than getting help from a friend (*M* = 3.77, *SD* = 1.50, *p* = 0.063, partial *η*^2^ = 0.012). However, there were no differences when the excluder was an immigrant (*M_teacherhelp_* = 3.52, *SD* = 1.40, *M_friendhelp_* = 3.41, *SD* = 1.52, *p* = 0.213, partial *η*^2^ = 0.005, see Figure 2). No other interactions were significant (all *p*s > 0.05). These findings indicate that youth favored getting help from a friend over a teacher or adult when the excluder was an ingroup peer (i.e., British). This bias to favor keeping bystander challenging as an internal peer group matter rather than involving teachers or other adults, however, was not evident when the excluder was an outgroup peer (i.e., an immigrant).

### Social and moral reasoning

Hypothesis 4 was not supported since moral reasoning was used less than 10% in the case of both forms of indirect bystander responding. However, there were differences between children and adolescents in terms of the type of social-conventional reasoning and the degree of psychological reasoning used to justify indirect bystander responses.

### Getting help from a teacher or an adult

The addition of predictors (Age Group, Excluder Membership, Victim Membership) to the model led to a significant improvement in the model fit compared to the null model (LR) *χ*^2^ (6, *N* = 172) = 46.91, Nagelkerke *R*^2^ = 0.269, *p* < 0.001. We observed a main effect of age group for getting help from a teacher or an adult, *χ*^2^ (3, *N* = 172) = 44.11, *p* < 0.001. Compared to adolescents, children were more likely to refer to their trust in teachers than group loyalty and dynamics, *β* = −2.37, *χ*^2^(1) = 28.32, *p* < 0.001, Exp (B) = 0.09, 95% CI [0.04, 0.22] and psychological reasons, *β* = −2.16, *χ*^2^(1) = 26.20, *p* < 0.001, Exp (B) = 0.11, 95% CI [0.05, 0.26] (see Table 4). For example, one child participant positively rated the getting help from a teacher or an adult item by referring to their trust in teachers: *“because teachers help you and if somebody is left out you can tell them and they fix it.”* Meanwhile, adolescents’ reasoning used notions of group dynamics and loyalty and psychological reasoning more than children. For example, adolescents justified their negative evaluations of getting help from a teacher or adult by referring to group dynamics and loyalty and said things like, “*it is best to sort it out between ourselves, teachers or adults might make the situation worse,*” or “*as I would not want my friends getting in trouble, I ain’t a snake.*” Finally, adolescents also used psychological reasoning like, “*I could sort it out myself*” more. There were no significant main effects of excluder membership, victim membership or any interactions (all *p*s < 0.05).

**Table 4:** Frequencies and proportions of participants’ reasoning of getting help from a teacher or an adult as a function of age group.

Age group	Trust in teachers	Group loyalty and dynamics	Psychological	Row total
Children	50 (0.65)	12 (0.15)	15 (0.20)	77
Adolescents	16 (0.17)	39 (0.41)	40 (0.42)	95
Column total	66	51	55	*N* = 172

Observed values are reported with proportions within group in brackets.

### Getting help from a friend

The addition of predictors (age group, excluder membership and victim membership) to the model led to a significant improvement in the model fit compared to the null model (LR) *χ*^2^ (6, *N* = 176) = 14.91, Nagelkerke *R*^2^ = 0.092, *p* = 0.021. We observed a main effect of age group on getting help from a friend, *χ*^2^ (2, *N* = 176) = 11.90, *p* = 0.003. Compared to adolescents, children were more likely to refer to their trust in friends than group dynamics, *β* = −1.20, *χ*^2^(1) = 8.23, *p* = 0.004, Exp (B) = 0.30, 95% CI [0.13, 0.68], and psychological reasons, *β* = −0.98, *χ*^2^(1) = 7.00, *p* = 0.008, Exp (B) = 0.37, 95% CI [0.18, 0.77] (see Table 5). For example, child participants positively rated getting help from a friend with reference to their trust in friends by reasoning that “*a friend will sort the problem out”* or “*friends are reliable.*” Meanwhile adolescent participants used group dynamics and loyalty and psychological reasoning more compared to children. For example, adolescents justified their likelihood of getting help from a friend by saying “*they may have the same perspective as [excluder]”* or “*it’s better if more people agree.”* Adolescent participants also referred to psychological reasons saying “*no one else should get involved”* or “*I can argue with them myself.*” There were no significant main effects of excluder membership or victim membership (all *p*s < 0.05).

**Table 5:** Frequencies and proportions of participants’ reasoning of getting help from a friend as a function of age group.

Age group	Trust in friends	Group loyalty and dynamics	Psychological	Row total
Children	42 (0.60)	11 (0.16)	17 (0.24)	70
Adolescents	36 (0.34)	31 (0.30)	39 (0.37)	106
Column total	78	42	56	*N* = 176

Observed values are reported with proportions within group in brackets.

The addition of the interaction term between age group and excluder membership, however, significantly improved the fit of the model, (LR) *χ*^2^ (6, *N* = 176) = 18.18, Nagelkerke *R*^2^ *= 0*.111, *p* = 0.006. The proceeding main effects of age group were qualified by this interaction term. Due to some small cell sizes, we followed the approach of other reasoning studies (e.g., McGuire et al., 2017) and conducted Fisher’s exact test and follow-up *z* tests with Bonferroni correction with multiple comparisons to investigate differences in participants’ reasoning to justify getting help from a teacher or an adult as a function of age group and excluder membership (means are proportional percentages of reasoning). The results showed that only when the excluder was British, children compared to adolescents were more likely to refer to trust in friends (*M* = 0.62) than group dynamics (*M* = 0.11, Fisher’s exact = 10.52, *p* = 0.005). However, there was no significant difference when the excluder was an immigrant (*p* = 0.06). For example, when the excluder was British, children referred to trust in friends more by saying, “*Because friends are really helpful*” or “*a friend helps*.” Meanwhile adolescents referred to group dynamics more by saying, “*I am not a snitch*” or “*they might be on your side*.”

The addition of the interaction term between age group, excluder membership and victim membership also significantly improved the fit of the model (LR) *χ*^2^ (14, *N* = 176) = 28.30, Nagelkerke *R*^2^ *=* 0.169, *p* = 0.013. The results showed that when both the excluder and victim were British, children were more likely to refer to trust in friendship (*M* = 0.74) more than group dynamics (*M* = 0.10) while adolescents referred to group dynamics more (*M* = 0.43) than trust in friends (*M* = 0.30, Fisher’s exact = 9.23, *p* = 0.011, see Table 6). For example, children positively rated getting help from a friend item by referring to trust in friendship, saying for example, “*you can trust friends*” or “*because they will help you and keep secrets*.” Whereas adolescents referred to group dynamics and loyalty more by saying, “*I would have more than one person on my side*.” There were no significant differences for other comparisons (all *p*s > 0.05).

**Table 6:** Frequencies and proportions of participants’ reasoning of getting help from a friend as a function of age group, the group membership of excluder, and the group membership of the victim.

Age group	Excluder membership	Victim membership	Trust in friends	Group loyalty and dynamics	Psychological	Row total
Children	British	British	14 (0.74)	2 (0.11)	3 (0.16)	19
		Immigrant	9 (0.50)	2 (0.11)	7 (0.39)	18
	Immigrant	British	8 (0.50)	5 (0.31)	3 (0.19)	16
		Immigrant	11 (0.65)	2 (0.12)	4 (0.23)	17
Total			42 (0.60)	11 (0.16)	17 (0.24)	70
Adolescents	British	British	9 (0.30)	13 (0.43)	8 (0.27)	30
		Immigrant	8 (0.36)	7 (0.32)	7 (0.32)	22
	Immigrant	British	11 (0.48)	2 (0.9)	10 (0.43)	23
		Immigrant	8 (0.26)	9 (0.29)	14 (0.45)	31
Total			36 (0.34)	31 (0.30)	39 (0.37)	106
Column total			78	42	56	*N* = 176

Observed values are reported with proportions within group in brackets.

## Discussion

In this study, we examined indirect prosocial bystander reactions to intergroup social exclusion, which are understudied but very crucial. We know how effective prosocial bystander reactions are in reducing bullying (Hawkins et al., 2001; Salmivalli et al., 2011) but children do not report prosocial bystander reactions often and their likelihood of engaging can decrease with age depending on the group membership of the victim and the perpetrator (Hawkins et al., 2001; Palmer et al., 2015; Gönültaş and Mulvey, 2020). Among the two types of bystander reactions (i.e., direct and indirect), indirect forms (e.g., intervening indirectly, without confronting bullies or drawing their attention) are important to examine because, compared to direct forms, they require less resources and risks (Levy and Gumpel, 2018; Lambe et al., 2019). In the current study, we explored developmental differences in children’s and adolescents’ indirect bystander reactions using hypothetical scenarios. We examined whether children and adolescents would get help from a teacher and get help from a friend when they witnessed a British or an immigrant peer being excluded by a British or an immigrant peer from a school club activity. We also investigated their reasoning about their likelihood of engaging in these indirect reactions.

Our results revealed novel developmental findings from middle childhood to adolescence. As predicted by our first hypothesis, participants’ likelihood of indirect bystander reactions decreased with age. In line with our second hypothesis, the findings revealed that while children preferred getting help from a teacher or an adult over getting help from a friend, adolescents were more likely to get help from a friend than getting help from a teacher or an adult. Our third hypothesis was partially supported. Participants were found to be marginally less likely to get help from a teacher and an adult than getting help from a friend only when the excluder was an ingroup peer, i.e., British but not when the excluder was an outgroup peer, i.e., an immigrant. The social and moral reasoning that this study examined also provided a novel insight into the developmental trends we found. For both indirect bystander reactions, children justified their likelihood of indirect intervention by referring to their trust in teachers and friends, while adolescents were more likely to refer to group loyalty and dynamics and psychological reasons.

The developmental decline we found in indirect hypothetical bystander reactions from childhood into adolescence is in line with previous research drawing from the Social Reasoning Developmental approach on bystander reactions to bullying in peer group contexts (Palmer et al., 2015; Mulvey et al., 2016; Gönültaş and Mulvey, 2020). We extended previous the Social Reasoning Developmental approach research on hypothetical bystander reactions to bullying (Palmer et al., 2015; Gönültaş and Mulvey, 2020) by showing that the developmental decrease in prosocial bystander reactions is also evident in the context of intergroup social exclusion. This finding fits with the Social Reasoning Developmental approach which indicates that from late childhood into adolescence, children’s evaluations and reasoning about social exclusion and bystander responses in peer group contexts increasingly pertain to their knowledge about peer group processes and group dynamics (Rutland et al., 2010). Having a more advanced understanding of peer group dynamics and considering increasing concerns about group-related and psychological factors, adolescents can become less likely to report indirect prosocial bystander responses with age.

The decreasing levels of getting help from teachers and friends from childhood into adolescence, however, is alarming since bullying, especially relational, indirect forms such as social exclusion, increases with age (Crick et al., 2002; Salmivalli and Peets, 2009). Moreover, teachers are not very adept in identifying relational and covert forms of bullying (Yoon and Kerber, 2003; Bauman and Del Rio, 2006) and they are less likely to identify bullying among adolescents compared to children (Leff et al., 1999; Yablon, 2017). In the case of social exclusion, which can be more subtle and ambiguous than other forms of bullying, this presents an additional challenge for teacher detection. The low likelihood of getting help from teachers and friends and the low likelihood of teachers identifying bullying prevent the victims from receiving the help and support they need.

Another novel finding from this study is that while children were more likely to get help from a teacher than getting help from a friend, adolescents were more likely to get help from a friend than from a teacher or an adult. The previous studies (e.g., Palmer et al., 2015; Gönültaş and Mulvey, 2020) did not fully capture this developmental trend as no study has examined age differences in these two indirect bystander responses to social exclusion separately. This finding indicates developmental differences in preferences regarding different forms of indirect bystander reaction. This can be explained by that getting help from a teacher and getting help from a friend can have different perceived group consequences for different age groups. The findings might suggest that with age, adolescents can become more aware of group processes such as group dynamics and group loyalty and the consequences of letting an authority figure know about the negative situation in general. This interpretation is in accord with research indicating that students think that teacher involvement in bullying situations can make things worse (Bradshaw et al., 2007; Boulton et al., 2017). Moreover, with age, children become more independent and their reasoning around bystander helping involves psychological concerns, i.e., autonomy and personal choice. This is also in line with previous research that showed that adolescents were more likely than children to use psychological reasons such as, “because it is not my business, I do not want to get involved” when they were asked to justify their reduced prosocial bystander intentions following incidents of verbal aggression (Palmer et al., 2015).

This study also extended previous research by identifying the effect of group membership on specific forms of indirect bystander reactions. Even though we did not find an effect for Hypothesis 3 in the expected main effects, there was a marginal effect for a related unhypothesized exploratory finding. Specifically, we found that participants were less likely to get help from a teacher or an adult than getting help from a friend only when the excluder was an ingroup peer, i.e., British. This finding might suggest that participants were concerned about being seen as disloyal to their ingroup by telling a teacher when the excluder was an ingroup peer. This finding is also in line with the Social Reasoning Developmental model in which group membership and group loyalty are considered important factors in peer groups that arise from an early age (Abrams and Rutland, 2008; Rutland et al., 2010; Misch et al., 2016). Children understand that as a member of their group, they are expected to be loyal to their group in order to be socially accepted and not excluded (Killen et al., 2013; Rutland et al., 2015). One piece of bystander research showed that when participants (8th and 10th graders) knew that the ingroup members supported a negative act (i.e., race-based humor), they thought that deviant peers who intervened to help the victim as a bystander were more likely to be excluded from the peer group, due to an increasing understanding of group dynamics (Mulvey et al., 2016).

The social and moral reasoning findings provided more insight into the developmental differences in participants’ likelihood of indirect bystander reactions. The results revealed that while children’s reasoning focused more on their trust in their teachers and friends more, adolescents focused more on group-related reasoning such as peer group loyalty and group dynamics as well as psychological reasons. This is a novel contribution to the literature emphasizing the importance of different social-conventional concerns in shaping indirect bystander reactions in childhood and adolescence. Previous bullying research has mainly focused on social-cognitive factors and perceptions (e.g., teacher attitudes, positive actions, positive relationship, perceived teacher/friend support, Evans and Smokowski, 2015; Jungert et al., 2016; Demol et al., 2020; Mulvey et al., 2020b) to explain indirect bystander reactions. These factors are important, however, might fail to capture the full picture. Bullying happens in peer groups and therefore peer-group-related factors such as group dynamics and group loyalty can also play an important role.

The current findings emphasize the increasing importance of group processes in adolescents’ indirect bystander reactions and reasoning. This supports the Social Reasoning Developmental approach, whereby as children develop increasing knowledge and understanding about the social world and group processes, with age, they start to weigh up different concerns (i.e., moral, group-related and psychological) when evaluating social exclusion and consequent bystander reactions (Killen and Rutland, 2011; Palmer et al., 2015). As a member of a peer group, they can develop a sense of belonging and loyalty to their groups and learn the dynamics of acting in accordance with their group membership, group norms and social norms in a wider perspective (Killen and Rutland, 2011; Killen et al., 2018). Future research should examine and manipulate group norms (i.e., the peer group helping or not helping victims) to further explain how they influence developmental trends in indirect bystander reactions and reasoning.

The reasoning findings also revealed decreasing levels of trust in teachers and friends with age. One qualitative study that examined the role of children’s perspectives of school staff support on their prosocial bystander reactions using semi-structured interviews found that students emphasized the importance of trust and safe relationships with teachers and school staff in their willingness to approach them (Wood et al., 2017). The reasoning findings from the current study support the previous evidence by showing the importance of trust as a social-conventional construct and extend it by showing how trust in teachers and friends changes developmentally from childhood into adolescence. Finally, the results showed increasing levels of psychological reasons used in participants’ justifications of their likelihood of indirect bystander reactions. This finding can be explained because as children get older, their sense of autonomy develops and they tend to deal with situations on their own instead of asking for help from others (Unnever and Cornell, 2004).

This study has some limitations. First, in this study, we examined participants’ hypothetical reactions (i.e., self-report measures), but not their actual bystander behavior. Although research shows that children’s hypothetical evaluations are in line with their actual bystander reactions (Mulvey et al., 2018), future research should use alternative methods such as social media simulations, virtual reality technologies or online game contexts (e.g., Yüksel et al., 2021). Future studies should explore how children and adolescents show indirect bystander behavior in real-life settings (see a review of methodological approaches in Palmer et al., 2021). Second, the current study is cross-sectional in nature. Future longitudinal studies would shed more light on how children’s indirect bystander behavior changes over time. Third, the order for the indirect bystander measures were not counterbalanced. Future research should consider this to control for any possible order effect. Fourth, we use single items to measure two different forms of bystander reactions. Future research should develop new items to measure the different forms of indirect bystander reactions to improve validity and reliability of the measures. Finally, there is a need for future research outside North America and Europe to examine the generalizability of these findings.

In sum, the present study provided novel developmental findings about children’s and adolescents’ indirect prosocial bystander reactions to social exclusion as well as the social and moral reasoning underlying their reactions. This study has important implications for research and school-based anti-bullying intervention programs (e.g., KiVA, Meaningful Roles) that focus on promoting prosocial bystander behavior to help reduce bullying in schools (Polanin et al., 2012; Salmivalli et al., 2012; Ellis et al., 2016). The current study highlights a developmental decline in reporting indirect prosocial bystander reactions from childhood into adolescence. We also demonstrate the importance of peer group dynamics and the intergroup context in determining indirect bystander responses to social exclusion. The finding that adolescents, compared to children, are more likely to speak to their peers than their teachers when they witness social exclusion suggests interventions should focus on normalizing bystander challenging in peer groups, so peers are more likely to act together to confront exclusion. Moreover, providing teachers with additional training on how to recognize social exclusion and how to intervene effectively can also be important as previous research has shown that teachers expressed a need for training in dealing with bullying situations (Bradshaw et al., 2013). Developmentally, making teachers more approachable and more understanding of why adolescents might not feel able to intervene can also be crucially important. Increasing teachers’ awareness around adolescents’ understanding of group-related concerns, social exclusion and their reactions to it (i.e., they can be less likely to intervene as they worry about being excluded themselves or they do not think they can make a difference) could help teachers to support adolescents’ well-being and self-efficacy. The effect of excluder membership also suggests that interventions need to focus on encouraging youth to indirectly challenge excluders by telling a teacher or adult, especially when the perpetrator is an ingroup peer. Overall, the key role of bystander interventions should be emphasized in schools and intervening as a bystander directly or indirectly to support the victim should be promoted to become a school and peer group norm.

## Data availability statement

The raw data supporting the conclusions of this article will be made available by the authors, without undue reservation.

## Ethics statement

The studies involving human participants were reviewed and approved by the Ethics Committee of Goldsmiths University of London. Written informed consent to participate in this study was provided by the participants’ legal guardian/next of kin.

## Author contributions

AY designed the study, developed the hypotheses, performed the data collection, analyzed the data, and drafted the manuscript. SP supervised the study design and helped to draft the manuscript. EA made contributions to the data analysis and helped to draft the manuscript. AR supervised the study design, oversaw the development of the hypotheses and statistical analyses, and helped to draft the manuscript. All authors contributed to the article and approved the submitted version.

## Funding

This work was supported by the Turkish Ministry of National Education Postgraduate Scholarship awarded to AY. Open access funding was provided by the University of Exeter.

## Conflict of interest

The authors declare that the research was conducted in the absence of any commercial or financial relationships that could be construed as a potential conflict of interest.

## Publisher’s note

All claims expressed in this article are solely those of the authors and do not necessarily represent those of their affiliated organizations, or those of the publisher, the editors and the reviewers. Any product that may be evaluated in this article, or claim that may be made by its manufacturer, is not guaranteed or endorsed by the publisher.
