# Supplementary material for: When do bystanders get help from teachers or friends? Age and group membership matter when indirectly challenging social exclusion
Source: Front Psychol. 2022 Aug 30;13:833589. doi: 10.3389/fpsyg.2022.833589 (PMC9468897; doi:10.3389/fpsyg.2022.833589)
Supplement: Supplementary file 1 [file Table_1.DOCX]

**Supplementary Material**

**Table S1 Repeated Measures ANOVA**

| *Within Subjects Effects* | | | | | | |  |
| --- | --- | --- | --- | --- | --- | --- | --- |
|  | Sum of Squares | df | Mean Square | F | p | |  |
| Factor | .48 | 1 | .48 | .22 |  | |  |
| Factor x age group | 84.99 | 1 | 84.99 | 39.10 | <.001 | |  |
| Factor x excluder membership | 10.22 | 1 | 10.22 | 4.70 | .03 | |  |
| Factor x victim membership | .72 | 1 | .72 | .33 | .56 | |  |
| Factor x age group x excluder membership | .09 | 1 | .09 | .04 | .83 | |  |
| Factor x age group x victim membership | .66 | 1 | .66 | .30 | .58 | |  |
| Factor x excluder membership x victim membership | .32 | 1 | .32 | .148 | .70 | |  |
| Factor x age group x excluder membership x victim membership | 1.21 | 1 | 1.21 | .55 | .45 | |  |
| Error | 619.46 | 285 | 2.17 |  |  | |  |
| *Between Subjects Effects* | | | | | | | |
|  | Sum of Squares | df | Mean Square | F | | p | |
| Intercept | 7495.50 | 1 | 7495.50 | 1879.88 | | <.001 | |
| Age group | 272.88 | 1 | 272.88 | 68.44 | | <.001 | |
| Excluder membership | .76 | 1 | .76 | .192 | | .66 | |
| Victim membership | 1.41 | 1 | 1.41 | .35 | | .55 | |
| Age group x excluder membership | 1.34 | 1 | 1.34 | .33 | | .56 | |
| Age group x victim membership | 8.73 | 1 | 8.73 | 2.19 | | .14 | |
| Excluder membership x victim membership | 6.60 | 1 | 6.60 | 1.65 | | .19 | |
| Age group x excluder membership x victim membership | 2.48 | 1 | 2.48 | .62 | | .43 | |
| Error | 1136.35 | 285 | 3.98 |  | |  | |

**Table S2 Regression Analyses**

| *Regression Analysis Summary (Getting help from a teacher or an adult)* | | | | | |
| --- | --- | --- | --- | --- | --- |
| Variable | B | 95% CI | β | t | p |
| (Constant) | 6.62 | [5.23, 8.02] |  | 9.33 | <.001 |
| Gender | .12 | [-.27, .51] | .03 | .61 | .54 |
| Intergroup contact | -.10 | [-.39, .17] | -.03 | -.74 | .45 |
| Age group | -2.17 | [-2.56, -1.78] | -.55 | -10.91 | <.001 |
| Excluder membership | .26 | [-.13, .65] | .05 | 1.31 | .19 |
| Victim membership | -.05 | [-.44, .33] | -.01 | -.26 | .79 |

Note: R^2^adjusted = .30. CI = confidence interval for B.

| *Regression Analysis Summary (Getting help from a friend)* | | | | | |
| --- | --- | --- | --- | --- | --- |
| Variable | B | 95% CI | β | t | p |
| (Constant) | 3.97 | [2.42, 5.51] |  | 5.06 | <.001 |
| Gender | .10 | [-.33, .53] | .03 | .47 | .64 |
| Intergroup contact | .26 | [-.05, .58] | .10 | 1.65 | .10 |
| Age group | -.66 | [-1.09, -.23] | -.18 | -3.02 | .003 |
| Excluder membership | -.20 | [-.63, .23] | -.05 | -.92 | .26 |
| Victim membership | .13 | [-.29, .56] | .03 | .62 | .53 |

Note: R^2^adjusted = .05. CI = confidence interval for B.
